# The impact of high fructose corn syrup on liver injury and glucose metabolism: a systematic review

**DOI:** 10.3389/fnut.2025.1724398

**Published:** 2025-11-26

**Authors:** Zane Z. Yu, Sneha Varahala, Sean L. C. Lim, Maimuna C. Marenah, Julia Wattacheril

**Affiliations:** 1 Mailman School of Public Health, Columbia University Irving Medical Center, New York, NY, United States; 2 Department of Internal Medicine, Warren Alpert Medical School, Brown University, Providence, RI, United States; 3 Center for Liver Disease and Transplantation, Columbia University—New York Presbyterian Hospital, New York, NY, United States

**Keywords:** liver injury, liver disease, high fructose corn syrup, metabolic health, MASLD

## Abstract

**Background:**

High fructose corn syrup (HFCS) is a dietary sweetener that is used in a substantial portion of food and beverages. Recent evidence has cited dietary HFCS as a risk factor in the development of obesity, metabolic syndrome, and liver disease. This systematic review provides a new evaluation of the potential hepatic and metabolic risks posed by HFCS to inform both clinical practice and public health policy.

**Methods:**

We conducted a systematic review of English-language, human studies of adults (≥18 years) with low to no alcohol intake using Covidence. Only quantitative studies that specified a link supported by direct evidence between HFCS and markers of liver injury or glucose metabolism in the setting of MASLD or NAFLD were included.

**Results:**

The literature search yielded 23,006 studies. After removing duplicates, 16,955 studies were screened and 16,930 were excluded after abstract screening. 20 texts were reviewed in full; 19 were excluded. 1 study was included after study selection.

**Conclusion:**

This review identifies and critically appraises the methodological strengths and limitations of the sole study meeting eligibility criteria. The 2022 study by Sigala et al. involved a non-randomized, controlled dietary intervention examining the dose–response effects of high-fructose corn syrup (HFCS) on hepatic lipid accumulation and insulin sensitivity in healthy adults. The identification of only a single eligible study emphasizes a stark absence of focused investigations in this area. Given the increasingly widespread consumption of HFCS and its prevalence in the modern food supply, this scarcity of research is concerning. Further research in this area should focus on clinical studies of longer duration, comparative studies of HFCS and other sugars, and incorporate greater demographic and geographic diversity.

## 1 Introduction

High fructose corn syrup (HFCS) has been repeatedly cited as a potential factor contributing to the increase in the prevalence of multiple pathologies over the years, including (but not limited to) obesity (1), diabetes (2), and kidney disease (3). HFCS differs from table sugar in its chemical composition. Unlike pure glucose, HFCS-55 is most commonly used in soft drinks, consisting of 55% fructose, 42% glucose, and 3% oligosaccharides (4). HFCS was first developed in 1968 in response to supply fluctuations and the resulting price instability of sugar (5). The development of this inexpensive sweetener led to rapid widespread adoption since 1987 and accounts for approximately 40% of all added caloric sweeteners in the US diet (6). Rising HFCS consumption has occurred in parallel with the increasing prevalence of metabolic syndromes, raising questions about the mediating role it plays in disease development (7).

Additionally, nonalcoholic fatty liver disease (NAFLD)—now known as metabolic dysfunction-associated steatotic liver disease (MASLD)—has been found to be the most common chronic liver disease in the Western world (8). As understanding of this disease classification advanced and recognition of its growing prevalence in public health at large progressed, there has been much discussion regarding the name. A recent consensus settled on the adaptation of a new definition, nomenclature, and classification scheme, resulting in the renaming of NAFLD to MASLD; the distinctions between the subcategories of this disease umbrella are not relevant to the scope of this paper (9). This systematic review was conducted with the broader criteria of diagnosing MASLD in mind; however, given the relative infancy of the term, the old nomenclature of NAFLD will be used interchangeably with MASLD in this paper.

The coinciding and increasing pervasiveness of HFCS across the food and beverage industry, and subsequently within everyday Western diets, has raised public health concerns about its scale and intensity of exposure to the average person. In particular, the use of HFCS in sugar-sweetened beverages (SSBs) have been associated with caloric overconsumption (1) positioning it as a significant modifiable dietary risk factor. As MASLD criteria includes all patients with steatosis and at least one of five cardiometabolic risk factors (broadly, high BMI, high fasting serum glucose, high blood pressure, high plasma triglycerides, or high plasma high-density lipoprotein) (9), dietary risk factors are, in turn, significant contributors to the growing prevalence of MASLD—now at about 38% of all adults globally (10).

Stanhope’s review on the link between sugar consumption and metabolic diseases showed strong support demonstrating both direct and indirect causation. However, gaps in definitive conclusions and industry-funded studies defending its safety have fueled this dietary controversy (11). Therefore, there is a need for ongoing reviews to evaluate whether the regulation of HFCS is justified in mitigating these adverse health outcomes.

While there are numerous well-documented studies highlighting the link between HFCS and obesity or diabetes, fewer studies analyze the specific effects on the liver as part of the metabolic pathway. HFCS promotes hepatocellular triglyceride accumulation and hepatic steatosis, both *in vitro* and *in vivo*, via increased lipogenesis and impaired insulin signaling. At the cellular level, HFCS exposure induces oxidative stress, mitochondrial dysfunction, and endoplasmic reticulum (ER) stress, accompanied by impaired calcium signaling and activation of other stress pathways. These disturbances result in insulin resistance, portal inflammation, and hepatocellular apoptosis, showing a potential pathway linking dietary HFCS consumption to the pathogenesis of non-alcoholic fatty liver disease (NAFLD) (12–15). Additionally, there was recently a study using a combined omics approach and murine data that pointed to HFCS as a significant contributor to steatosis aggravation in obesity-related NAFLD (16).

A previous systematic review by Chung et al. assessed the effects of multiple dietary sugars (fructose, sucrose, and HFCS) on various liver metabolic and necro-inflammatory markers such as hepatic lipid content and liver enzyme levels (ALT, AST, γ-glutamyl transpeptidase) (17). However, their analysis grouped multiple sugar types together and relied solely on indirect comparisons. Notably, Chung et al. also combined both adult and pediatric populations (> = 4 years), which may introduce the possibility of confounding, given significant physiological and metabolic differences between both populations. While Chung’s study focused broadly on dietary fructose and its impact on liver health, the role of HFCS specifically on the liver was uncertain. In another systematic review, Gopalakrishnan Ravikumar et al. (18) focused specifically on HFCS, but reviewed purely murine studies, limiting its applicability to human populations.

In addressing these limitations, this systematic review explicitly isolates HFCS as a distinct dietary exposure, specifically targeting adult human populations (> = 18 years) to reduce heterogeneity and enhance metabolic comparability. The aim of this study is to evaluate the association between HFCS consumption and markers of liver injury—which, per Thakur et al. (19) will include AST, ALT, ALP, bilirubin, GGT, and PT/INR as clinical detectors of liver disease and indicators for extent of liver damage—and glucose metabolism in human adults, synthesizing evidence from original, quantitative studies to provide an updated assessment of the potential hepatic and metabolic risks posed by HFCS and ultimately inform both clinical practice and public health policy.

## 2 Methods

### 2.1 Sources of information

**Figure 1:**
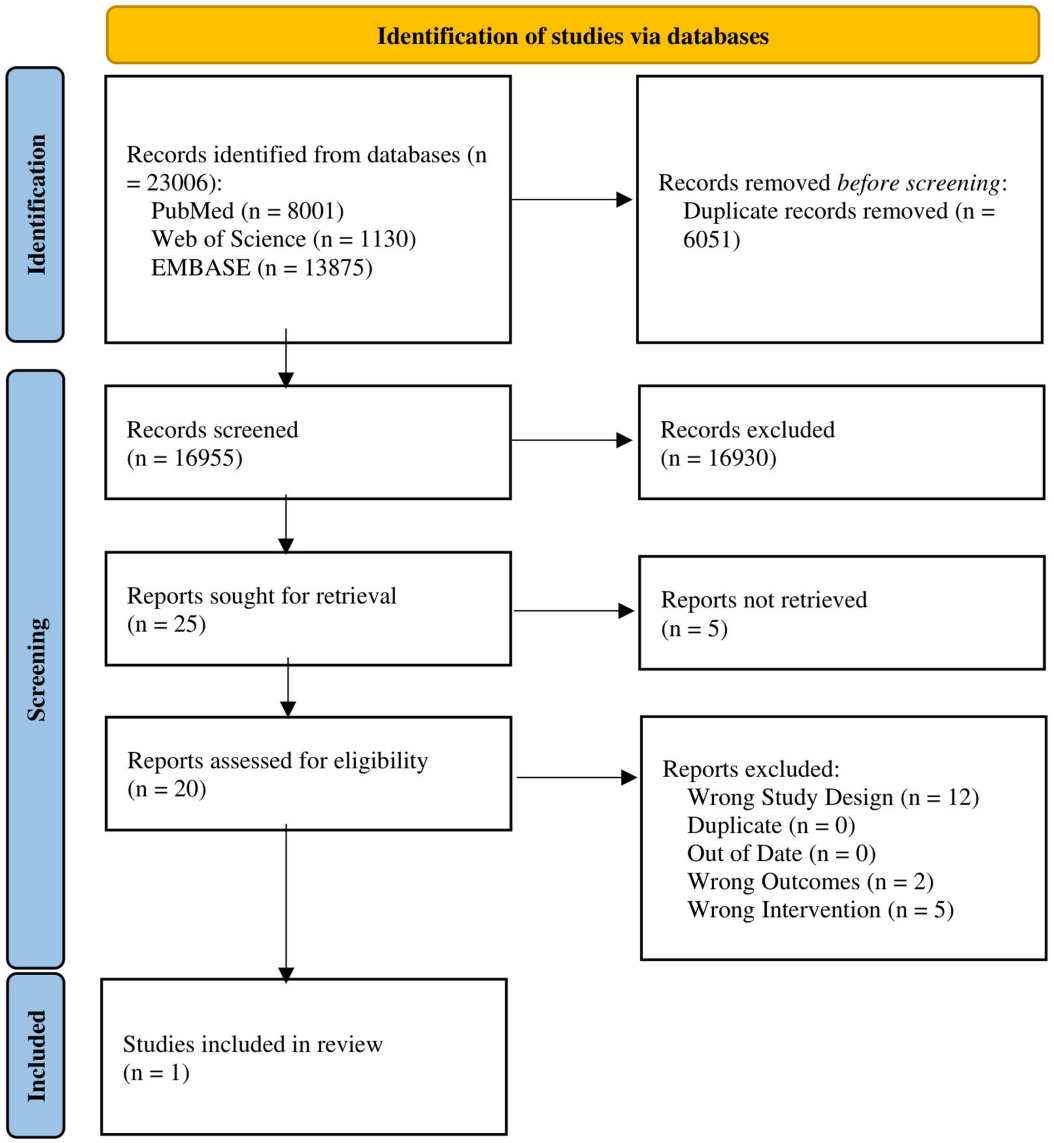
PRISMA flow diagram.

In accordance with the Preferred Reporting Items for Systematic Reviews and Meta-Analyses (PRISMA) guidelines (20), we created a comprehensive search strategy (Figure 1). We identified studies from three different electronic databases: PubMed, Web of Science, and EMBASE. Searches were conducted between October 2024 and November 2024. Each of these databases was last consulted on November 1st, 2024.

### 2.2 Eligibility criteria

The eligibility criteria was drafted according to the guidelines posed by the Cochrane Handbook for Systematic Reviews of Interventions (21). Eligibility criteria included peer-reviewed, English-language studies that specified a link supported by direct evidence between HFCS and markers of liver injury or glucose metabolism in the setting of MASLD or NAFLD. Studies must have been published any time earlier than November 1st, 2024. We excluded studies that analyzed solely HFCS or solely markers of liver injury or glucose metabolism. We also included all studies that focused on HFCS as it pertains to markers related to both liver injury and glucose metabolism in the setting of MASLD or NAFLD. We excluded studies focusing only on non-HFCS sweeteners (aspartame, sucrose, xylitol, etc.). Studies must have been based on adult human subjects (age greater than or equal to 18) with low to no alcohol intake in order to isolate HFCS effects and with no prior conditions that inherently affected fructose metabolism. Studies needed to be original and quantitative in nature, using specific biomarkers as metrics for quantifying liver injury and glucose metabolism. We excluded all qualitative studies and other systematic reviews or non-primary research types.

### 2.3 Search method

A grey literature search was conducted as an initial scoping review to assess any recent systematic reviews in this area or any explicitly pertinent studies that met inclusion criteria. The search string used was “high fructose corn syrup liver glucose”; the database used was Google Scholar. Grey literature search was performed by ZZY and SV on October 17th, 2024. Of note, all studies isolated by the initial grey literature search have either been cited in this paper or were included in the formal database search.

Our complete formal search strategy is detailed in Appendix 1. Our searches were specific to each database, prioritizing Medical Subject Headings (MeSH) terms and Emtree indices where appropriate. Each search string was separated into two concepts: liver disease (non-alcoholic) and high fructose corn syrup. The search string for each concept was then further supplemented by the addition of relevant title and abstract strings to address gaps that may not have been addressed by MeSH terms or Emtree indexing.

*Study selection*. Eligible studies were de-duplicated in EndNote X9.3.3 (Clarivate Analytics, Philadelphia PA, USA) using the method described by Bramer et al. (22) and imported into the systematic review software Covidence (Melbourne, Victoria, Australia) for screening, full-text review, and data extraction. Covidence was then used to de-duplicate a second time. Three reviewers (SLL, MCM, ZZY) conducted abstract screening and full-text review independently and in duplicate. One reviewer (ZZY) conducted screening and review for all eligible studies. The other two reviewers (SLL, MCM) conducted screening and review for half of all eligible studies each. Disagreements were arbitrated by the senior author (JW).

### 2.4 Data collection and risk of bias assessment

A data extraction template developed in Covidence was used to extract relevant information, including study design, baseline values, and outcome values. Two reviewers (SLL, ZZY) completed data extraction independently and in duplicate. Discrepancies were resolved by discussion and consensus. Two reviewers (MCM, ZZY) manually assessed risk of bias of the included study independently and in duplicate through the use of the Risk Of Bias In Non-randomized Studies—of Interventions, Version 2 (ROBINS-I V2) tool for each of the five primary outcomes. The tool consists of 7 domains to address quality and risk of bias in non-randomized interventional studies and is the preferred tool for evaluation of non-randomized studies for interventions per the Cochrane Handbook for Systematic Reviews of Interventions (23, 24). Discrepancies were resolved by discussion and consensus. One author (SV) performed an integrated risk of bias assessment.

### 2.5 Approach to analysis

Given the inclusion of a single eligible study, no meta-analysis was conducted. Instead, a structured qualitative synthesis was performed to evaluate the direction, strength, and consistency of the reported outcomes across predefined liver and metabolic markers.

## 3 Results

### 3.1 Study selection

Of 23,006 studies yielded by the literature search, 5,907 duplicates were removed through EndNote by the method detailed by Bramer et al. (22) and 144 duplicates were removed by Covidence. A total of 16,955 studies were screened; 16,930 were excluded after abstract screening. Full texts for 5 studies were unable to be retrieved. 20 texts were reviewed in full; 19 were excluded; 1 study was included (Figure 1) (25).

### 3.2 Outcomes

Study outcomes are detailed in Table 1.

**Table 1:** Study outcomes.

Outcome	Aspartame (*n* = 23)	10% HFCS (*n* = 18)	17.5% HFCS (*n* = 16)	25% HFCS (*n* = 28)
Baseline				
Hepatic lipid content (MRI-PDFF, %)	1.6 ± 0.8	1.2 ± 0.2	1.4 ± 0.3	2.3 ± 0.8
Predicted M ISI (arbitrary units)	1.52 ± 0.06	1.54 ± 0.06	1.60 ± 0.06	1.52 ± 0.05
Matsuda ISI (arbitrary units)	3.6 ± 0.3	3.7 ± 0.2	3.5 ± 0.2	3.3 ± 0.2
OGTT glucose AUC (mmol/L × 3 h)	20.9 ± 0.7	19.2 ± 0.8	19.1 ± 1.0	21.1 ± 0.7
OGTT insulin AUC (pmol/L × 3 h)	1392 ± 184	1033 ± 65	1292 ± 162	1335 ± 106
HOMA-IR (arbitrary units)	3.0 ± 0.2	3.0 ± 0.3	2.5 ± 0.2	3.0 ± 0.2
Lactate AMP (mmol/L)	0.68 ± 0.06	0.62 ± 0.06	0.69 ± 0.06	0.73 ± 0.06
Glucose AMP (mmol/L)	1.77 ± 0.19	1.88 ± 0.18	1.90 ± 0.22	2.04 ± 0.18
Insulin AMP (pmol/L)	544.5 ± 84.0	424.3 ± 46.5	525.0 ± 62.5	506.9 ± 45.1
Body weight (kg)	71.8 ± 2.2	70.9 ± 2.4	69.9 ± 3.6	72.9 ± 2.7
Intervention				
Hepatic lipid content (MRI-PDFF, %)	1.4 ± 0.7	1.3 ± 0.2	1.6 ± 0.3	2.8 ± 0.9
Predicted M ISI (arbitrary units)	1.55 ± 0.05	1.54 ± 0.05	1.56 ± 0.06	1.45 ± 0.07
Matsuda ISI (arbitrary units)	3.9 ± 0.3	3.5 ± 0.3	3.2 ± 0.3	3.0 ± 0.3
OGTT glucose AUC (mmol/L × 3 h)	19.9 ± 0.7	19.7 ± 0.9	20.3 ± 1.2	22.5 ± 0.9
OGTT insulin AUC (pmol/L × 3 h)	1229 ± 151	1188 ± 97	1376 ± 165	1625 ± 154
HOMA-IR (arbitrary units)	2.9 ± 0.2	2.9 ± 0.3	2.8 ± 0.2	3.3 ± 0.3
Lactate AMP (mmol/L)	0.73 ± 0.04	1.01 ± 0.07	1.50 ± 0.11	1.60 ± 0.09
Glucose AMP (mmol/L)	1.87 ± 0.19	2.30 ± 0.16	2.63 ± 0.18	2.96 ± 0.21
Insulin AMP (pmol/L)	567.4 ± 75.7	450.7 ± 35.4	588.9 ± 54.2	595.9 ± 48.6
Body weight (kg)	71.7 ± 2.2	70.9 ± 2.4	70.2 ± 3.7	73.7 ± 2.8

## 4 Analysis

### 4.1 Critical appraisal of methods

- *Design and allocation*: this was a non-randomized, parallel-arm intervention trial. Participants were allocated to groups by matching with respect to sex, BMI, and baseline metabolic parameters (fasting lipids, glucose, and insulin). While baseline characteristics were indeed comparable, the lack of randomization presents a potential confounding bias, as unevaluated variables might differ between groups. As reported by the authors, the absence of random allocation is a key limitation.
- *Blinding*: the trial was fully double-blinded. Beverages for each group were prepared centrally by staff not involved in direct care or assessments. Non-caloric and HFCS beverages were identically flavored (0% drinks contained aspartame plus flavoring; HFCS drinks had flavoring only) to mask taste differences. Study coordinators and nurses serving meals were blinded to assignment. Outcome assessors (MRI technicians, lab analysts) were also blinded. This robust blinding reduces both performance and detection bias.
- *Dietary control*: inpatient diet was tightly controlled: low added-sugar standardized menus for days 1–3 (baseline) and isoenergetic menus for days 18–19 with HFCS substitution. However, outside the clinical unit (days 4–17), participants consumed their usual ad libitum diets with study beverages. This outpatient phase is a strength in ecological validity but a limitation in control: total sugar and calorie intake from other sources could not be fully quantified. Participants were instructed to maintain usual diet and activity, and weight was monitored; nonetheless, free-living conditions introduce variability.
- *Intervention fidelity and compliance*: beverage dosing was precisely based on individual energy requirements. Compliance was vigilantly monitored using a riboflavin tracer (0.015 mg/mL in all beverages). Urinary riboflavin was measured on days 8, 12, and 17–20 to verify intake. Riboflavin levels remained similar between inpatient and outpatient periods and across groups, indicating high and comparable adherence. Notably, participants were informed of the tracer, which likely encouraged drinking all beverages; however, awareness of the marker could also potentially alter behavior. Only one participant discontinued—the reason was cited as “family emergency”—and there were a few missed final tests, suggesting minimal attrition.
- *Measurements*: *Hepatic fat* was quantified by MRI proton density fat fraction (PDFF) using validated imaging software. *Insulin sensitivity* was estimated by the Matsuda Insulin Sensitivity Index (M-ISI, 2-h OGTT) and Predicted Matsuda Insulin Sensitivity Index (Predicted M-ISI, 2-h OGTT with BMI adjustment). OGTT blood samples (glucose, insulin) were collected at 0, 30, 60, 90, 120, and 180 min; glucose was measured enzymatically (YSI analyzer); and insulin was measured by RIA. Postprandial plasma *lactate* and *glucose* were measured via YSI analyzer alongside glucose. Triglycerides, uric acid, and apoC-III were assayed from pooled 24-h blood collections. All assays report low coefficients of variation. Two coders (statistician and PI) supervised analysis, enhancing reliability. Overall, measurement methods were prudent and applied uniformly, yielding low risk of measurement bias.

### 4.2 Results interpretation

- *Hepatic lipid content*: HFCS dose produced a clear linear increase in liver fat over 2 weeks. At baseline, MRI-PDFF was low and similar across groups (means = approx. 1–2%). After intervention, mean PDFF rose in a dose-dependent fashion (Table 1), with a significant linear trend (dose *p* = 0.016). The highest (25%) group showed the largest absolute increase. The dose–response relation indicates even 10–17.5% of Ereq from HFCS modestly elevated hepatic fat relative to control.
- *Insulin sensitivity (OGTT indices)*: both OGTT-derived indices worsened with HFCS dose (Table 1). These trends were statistically significant (dose *p* = 0.0087 for M-ISI; *p* = 0.0072 for Predicted M-ISI). In pairwise terms, the 25% HFCS group had significantly lower insulin sensitivity than the 0% group (Table 1). Notably, HOMA-IR did not change significantly (*p* = 0.11), suggesting that dynamic OGTT measures were more sensitive to early insulin resistance.
- *Glucose and Insulin AUC*: post-OGTT glucose AUC increased in dose-dependent manner with HFCS (Table 1). This trend was statistically significant (*p* < 0.0004). Baseline 3-h glucose rose modestly in HFCS groups. Insulin AUC rose even more dramatically. This hyperinsulinemic response can be interpreted as a deterioration of insulin sensitivity.
- *Mechanistic biomarkers*: Postprandial lactate responses increased markedly in HFCS groups. The mean post-meal lactate “amplitude” (peak minus nadir) rose from ~0.68 mmol/L at baseline to 1.50–1.60 mmol/L in the 17.5–25% groups, compared to 0.73 mmol/L in controls. Lactate AMP showed a highly significant dose effect (*p* < 0.0001). Similarly, post-meal glucose AMP rose with dose (also *p* < 0.0001). Sigala et al. (26) report that increases in lactate mediated much of the insulin-sensitivity decline: statistical models showed that the rise in lactate AMP explained ~71% of the HFCS effect on M-ISI (and ~36–38% of the effect on OGTT insulin/glucose AUC). In other words, HFCS likely promotes insulin resistance partly via increasing lactate (a fructose metabolite). Uric acid and triglyceride changes were reported in Sigala et al.’s (26) prior publication, confirming known fructose effects.
- *Other outcomes*: there were no significant changes in body weight in the short term, despite some group differences in mean weight (25% HFCS group ended ~1 kg heavier on average). This suggests the metabolic effects occurred largely independent of weight gain. No adverse events were reported. All primary outcomes were robust to adjustment for sex and a metabolic risk score based on metabolic syndrome risk factors (MSRF).

Overall, the data show clear dose–response relationships: higher HFCS intake produced greater increases in liver fat and greater reductions in insulin sensitivity (25). The statistical analysis used ANCOVA with baseline adjustment; reported *p*-values for the HFCS dose effect were strong (all ≤0.016 for key outcomes). The trends were monotonic across the range 0–25% of Ereq, supporting a causal interpretation within the experimental context.

### 4.3 Risk of bias

Summaries of risk of bias assessments per ROBINS-I V2 are detailed in Table 2.

**Table 2:** Summary of risk of bias assessments per ROBINS-I V2.

Outcome	Confounding controlled?	Adjustment appropriateness	Risk of bias	Notes
OGTT glucose AUC	PY	No negative controls or over adjustment	*Low*	Good control of sex, BMI, baseline glucose/lipids. No post-intervention over adjustment.
OGTT insulin AUC	PY	No	*Low*	Same as glucose AUC. No signs of inappropriate adjustment.
Predicted Matsuda index (predicted M-ISI)	PY	No	*Low*	Matched groups, good control of confounding.
Matsuda index (M-ISI)	PY	No	*Low*	Controlled for key variables, but not all mechanistic factors.
MRI-PDFF (liver fat)	PY	No	*Critical*	12% missing; no imputation or dropout analysis; violates ROBINS-I guidance for complete-case analysis; final sample = 75/85.

“PY” (Partial Yes) reflects moderate confidence in confounding control, but not full certainty.

Per the ROBINS-I V2 tools, a “low” risk of bias to implies little or no concern about bias. A “critical” risk of bias implies that the study is very problematic, where characteristics of the study give rise to a critical of bias in the result such that the result should generally be excluded from evidence syntheses (23).

### 4.4 Integrated risk of bias assessment

Summaries of integrated risk of bias assessments are detailed in Table 3.

- *Confounding (pre-intervention)*: *moderate risk.* Without randomization, residual confounding cannot be excluded. The investigators matched groups on obvious confounders (sex, BMI, lipids, insulin) and blinded staff, strengthening internal validity; additionally, baseline characteristics were similar, which mitigates known confounding. However, unmeasured factors (e.g., dietary habits, gut microbiota, physical activity, body composition, genetics) may still differ by group. The relatively large number of potential confounders controlled (age, diet limitations, physical activity) helps, but the non-random allocation inherently allows for potential allocation bias.
- *Selection of participants*: *low-to-moderate risk.* All eligible volunteers who met strict inclusion/exclusion criteria were enrolled, reducing selection bias. Attrition was minimal (1 drop-out, a few missing data points). However, participants were self-selected, healthy young adults; this limits generalizability but does not bias internal comparisons. The one withdrawal (in the 17.5% group) was unrelated to intervention and follow-up rates were similar across groups, suggesting balanced selection.
- *Classification of interventions*: *low risk.* Intervention groups were clearly defined by HFCS dose (0, 10, 17.5, and 25% of Ereq). This allowed assessment of linear trends, which adds mechanistic insight and statistical power. The clear dose-dependent trends beyond what could attributed to group differences strengthen causal inference. No participants crossed over between arms. Beverages were labeled by code, but actual HFCS content was fixed and not subject to misclassification.
- *Deviations from intended interventions*: *low risk.* The use of a riboflavin biomarker (and participant awareness thereof) yielded near-100% adherence, which is rare in free-living dietary studies. This makes the reported dose–response effects highly credible (they are unlikely to be due to non-compliance). However, the use of a riboflavin biomarker might also introduce behavior bias. Knowing that urine would be checked could have motivated higher adherence or other diet changes not representative of real-world behavior. Moreover, riboflavin itself (a vitamin) may conceivably affect metabolism, though the dose was small. These “open-label compliance” factors are not discussed by the authors but warrant caution. Co-interventions (other sugar intake) were supposed to be unchanged; participants were instructed not to alter sugar habits and remained weight-stable. The controlled inpatient diets also minimized deviations. The lack of randomization here does not translate into differing adherence between groups, as compliance markers were similar. Thus, deviations likely had minimal bias (however, free-living diet variance is noted as a limitation).
- *Missing data*: *critical risk.* Group sizes were modest (*n* = 15–28). For the MRI-PDFF outcome, 12% of participants (10 of 85) were reported as having missing data. However, the breakdown provided in the manuscript accounts for 11 individuals: one participant could not tolerate the scan, one scan file was corrupted, and nine were attributed to scanner malfunction or scheduling failure. While this inconsistency may reflect a simple reporting oversight, it also introduces ambiguity in participant flow and undermines confidence in attrition accounting. The study does not report any imputation, dropout comparison, or sensitivity analyses, nor is a complete-case approach explicitly justified. According to ROBINS-I V2 guidelines, the lack of analysis to address >10% missing outcome data constitutes critical risk of bias, particularly when the lack of data could plausibly affect the outcome. OGTT Predicted M-ISI could not be calculated for 6 subjects due to missing samples. The burden of missing values appears balanced (spread across groups) and was handled via intention-to-treat ANCOVA. The authors do not report any sensitivity analysis for missing data, though the impact is tentatively small given the otherwise low dropout rate. No confidence intervals are reported for main outcomes, so the uncertainty bounds are unclear. Given the absence of a complete-case analysis justification and the proportion of missing data, this outcome is rated at critical risk of bias.
- *Measurement of outcomes*: *low risk.* All outcomes were measured with validated, objective methods by blinded personnel. MRI and lab assays have high precision; indices were calculated uniformly. The blinding of assessors prevents differential measurement bias. The use of multiple insulin-sensitivity metrics (M-ISI, Predicted M-ISI, HOMA-IR) provides corroboration. Although assessors were reportedly blinded for MRI, insulin, and lactate analysis, no explicit mention of blinding for OGTT glucose measurement was provided, despite it being measured using a YSI analyzer. This inconsistency does not necessarily imply bias but does reflect incomplete reporting across outcomes.
- *Selection of the reported result*: *low risk.* The study appears to report all pre-specified primary and key secondary outcomes (liver fat and insulin sensitivity measures). The ClinicalTrials.gov listing (2008 registration) aligns with published outcomes. There is no obvious outcome-switching, and the authors transparently report both significant and non-significant results (e.g., HOMA-IR). One caveat is that some mechanistic data (e.g., detailed triglyceride and uric acid time courses) are placed in prior or supplementary publications. Nonetheless, the main analyses are fully reported. Raw individual data were not provided publicly, but summary results are comprehensive.

**Table 3:** Summary of integrated risk of bias assessment.

Domain	Judgment	Rationale
Confounding	*Moderate*	Matching used instead of randomization; no random assignment; limited unmeasured confounding control.
Selection of participants	*Low to Moderate*	Minimal attrition; one withdrawal; eligibility criteria strict.
Classification of interventions	*Low*	HFCS dose clearly assigned; blinded delivery; good group labeling.
Deviations from intervention	*Low*	Riboflavin biomarker verified compliance; ad libitum diet uncontrolled but likely not differential.
Missing data	*Critical*	MRI and OGTT data partially missing (*n* = 71 analyzed vs 85 enrolled). MRI-PDFF 12% missing, no imputation or complete-case justification.
Outcome measurement	*Low*	Objective methods (MRI, OGTT); blinded assessors. Blinded, validated MRI; incomplete blinding statement for glucose noted.
Selective reporting	*Low*	Pre-specified outcomes reported; no evidence of outcome switching.

Similar to definitions set by the ROBINS-I V2 tools, we understood a “low” risk of bias to imply little or no concern about bias. A “moderate” risk of bias implies that there is some concern about bias in the result, although it is not clear that there is an important risk of bias. A “critical” risk of bias implies that the study is very problematic, where characteristics of the study give rise to a critical of bias in the result such that the result should generally be excluded from evidence syntheses.

## 5 Discussion

This systematic review critically appraises the methodological strengths and limitations of the sole study meeting eligibility criteria for this systematic review – the 2022 study by Sigala et al. (25) a non-randomized, controlled dietary intervention examining the dose–response effects of high-fructose corn syrup (HFCS) on hepatic lipid accumulation and insulin sensitivity in healthy adults. While the trial delivers robust experimental evidence regarding the short-term metabolic consequences of HFCS exposure, several design limitations (particularly those pertaining to missing data, lack of randomization, and incomplete dietary control) warrant careful consideration when interpreting the findings.

A key strength of the study lies in its use of validated, high-sensitivity, objective outcome measures (MRI-PDFF, OGTT-based insulin sensitivity indices), a dose-ranging intervention design, and biochemical compliance verification using riboflavin tracers. The trial demonstrated statistically significant and dose-dependent increases in hepatic lipid content alongside declines in insulin sensitivity with as little as 10–25% of daily energy intake from HFCS. These effects were observed over a short 15-day period, underscoring the potential for rapid metabolic deterioration in response to added HFCS.

Exploratory mediation analyses further implicated uric acid and lactate elevations as mechanistic intermediates, aligning with existing preclinical literature on fructose-induced hepatic stress and insulin resistance (3, 7, 26). The study’s rigorous design and objective endpoints lend credibility to the conclusion that HFCS is a causal contributor to early NAFLD and insulin resistance risk factors.

Nonetheless, there are important caveats inherent to the interpretation and extrapolation of these results. The study’s non-randomized design and short follow-up duration mean that long-term effects and population-wide implications remain uncertain, limiting the causal inference that can be drawn. The cohort was limited to healthy, young–middle-aged adults with normal BMI. Findings may not extend to older individuals, children, or those with obesity/metabolic syndrome. Effects of HFCS could differ by age, adiposity, or insulin resistance status. Although partly free-living, the study setting involved daily meal visits and prepared beverages, which differs from natural consumption. Palatability or satiety effects might not mirror real-life HFCS intake (e.g., beverages were unflavored Kool-Aid-style rather than commercial sodas). This may limit ecological generalizability. The HFCS “doses” tested – up to 25% of daily caloric intake—may exceed average consumption levels, also potentially limiting generalizability. Additionally, background dietary intake and lifestyle behaviors were not fully controlled during the outpatient phase. For clinical or policy guidance, these findings should be considered alongside larger-scale epidemiologic data and longer-duration trials. Critically, the study underscores that dose matters; even incremental increases in HFCS consumption may produce meaningful metabolic consequences.

## 6 Limitations of the systematic review

This study has several limitations. The immediate pitfall is the inclusion of only one study that met all inclusion and exclusion criteria. This effectively limits the generalizability of the findings and does not allow any room for conclusions regarding data beyond the study we have included (25). This further limits the external validity of our study. Additionally, we were unable to perform a meta-analysis. Any conclusion drawn from the data at hand is subject to the biases already present in Sigala et al. (25). It is difficult to make concrete judgements or recommendations regarding policy through a single study, and this systematic review is thus subject to criticisms regarding both prematurity and redundancy.

## 7 Conclusion

This systematic review, through Sigala et al. (25) provides some experimental evidence linking HFCS consumption to early markers of metabolic dysregulation in healthy young adults. Over a two-week intervention, participants consuming beverages containing increasing doses of HFCS (0, 10, 17.5, 25% of daily energy requirement) demonstrated clear dose–response relationships across key metabolic endpoints. Notably, higher HFCS intake was associated with significantly increased hepatic lipid accumulation, measured objectively by MRI-PDFF, and diminished insulin sensitivity, as captured by both Matsuda and Predicted M indices derived from OGTTs (25). These metabolic changes occurred even with the controlled nature of numerous other variables, including age, BMI, and fasting total cholesterol.

From a public health and clinical standpoint, the implications are nontrivial. Sigala et al. provide strong experimental evidence that even moderate consumption of HFCS-sweetened beverages can produce rapid, adverse metabolic effects in healthy adults. These effects appear within 2 weeks and occur independently of weight gain; this suggests that HFCS may contribute to early pathophysiologic changes preceding possible metabolic disease and may carry implications for ongoing injury and metabolic derangement with sustained exposure. While the study alone does not draw any conclusions regarding long-term outcomes or clinical risk thresholds, it does contribute to an expanding corpus of literature that links sugar-sweetened beverages to liver dysfunction and glucose metabolism (12). As our dietary patterns continue to evolve and regulatory bodies reconsider limits on added sugars, studies like this play a pivotal role in informing thresholds dictating safe intake along with identifying metabolic cutoffs that may precede disease onset.

Despite its narrow evidentiary base, this systematic review aims to assess what is clearly a critical void in existing literature regarding the isolation of HFCS as a specific dietary exposure and the evaluation of its association with hepatic and metabolic dysfunction in human adults. The identification of only a single eligible study – despite an exhaustive search strategy—emphasizes a stark absence of focused investigations in this area. Given the increasingly widespread consumption of HFCS and its entrenchment in the modern food supply (6), this scarcity of research is both surprising and concerning.

Additionally, throughout the screening process, there were multiple studies that met many, but not all, of the inclusion and exclusion criteria. A preponderance of such studies relied on the assessment of sugar-sweetened beverages (SSBs) and their effects on the liver and glucose metabolism (27–29); however, these studies did not make the distinction between sugar types. There were a few studies that did examine the link between specific sugars and metabolic dysfunction, but they would focus exclusively on fructose (30) or sucrose (31) alone. In other words, greater attention must be granted to the specific effects wrought by high fructose corn syrup on health. Notably, the authors had published another study focusing on fructose, glucose, HFCS, sucrose, and aspartame using the same study data, ultimately isolating fructose-sweetened beverages as the common denominator in negatively impacting hepatic insulin sensitivity in young adults (32).

Future work ought to prioritize randomized controlled trials of longer duration and greater demographic and geographic diversity to better characterize the effects of HFCS (acute or chronic) on both liver health and glucose metabolism. Comparative studies which assess HFCS against other commonly consumed sugars, like sucrose or glucose, would help identify and specify the metabolic risks that may be unique to HFCS. These projects should—as did Sigala et al. (25)—revolve around use of validated and objective outcome measures. In the context of evaluating MASLD and hepatic injury, it would be prudent to broaden the number of assayed metrics to include common clinical values: this would entail documentation of liver enzymes (aspartate aminotransferase, alanine aminotransferase, gamma-glutamyl transferase), liver proteins (albumin, total protein), metrics evaluating liver synthetic function (prothrombin time, partial thromboplastin time), and other values (including bilirubin) (33). These future studies should also account for potential missing data discrepancies by performing multiple imputation to bolster results. While some of these issues may be alleviated by the oft-proposed goal of enrolling more study participants, the logistics of acquiring more data, funding, enrollees, etc. are understandably complex; in these cases, and where multiple imputation may fail or be deemed inappropriate, a complete-case analysis would be indicated (34), especially in elimination of risk of bias. Until such data is available, clinicians and policymakers must interpret existing findings with caution and remain alert to a growing body of evidence that suggests the causal role of HFCS in the pathogenesis of early metabolic disease (1–3, 12, 25, 26). The urgency of further investigation cannot be overstated, particularly as metabolic dysfunction continues to rise globally amidst persistent exposure to added sugars (10). Multiple studies in recent years have already isolated HFCS as a likely driving factor behind kidney disease (3), pediatric asthma (35), and cardiac injury (36); its influence on metabolism and hepatic function should be interrogated now more than ever if the projected 55% prevalence of MASLD in adults by 2040 (10) is to be avoided.

Sigala et al. (25) present a carefully controlled, dose-ranging trial demonstrating that HFCS-sweetened beverage consumption has graded adverse effects on liver fat and insulin sensitivity in healthy adults. This review acknowledges the strong methodology and insightful findings, while noting design limitations. Continued research building upon this work will conceivably help translate these findings into robust dietary recommendations and clinical insights.

## Data Availability

The original contributions presented in the study are included in the article/Supplementary material, further inquiries can be directed to the corresponding author/s.
