# Supplementary material for: The impact of high fructose corn syrup on liver injury and glucose metabolism: a systematic review
Source: Front Nutr. 2025 Nov 26;12:1724398. doi: 10.3389/fnut.2025.1724398 (PMC12689413; doi:10.3389/fnut.2025.1724398)
Supplement: Supplementary file 1 [file Table_1.docx]

**Appendix 1:** Search strategy

| **Database** | **Search Query** |
| --- | --- |
| PubMed | ( "liver diseases"[MeSH] OR "fatty liver"[MeSH] OR "liver failure"[MeSH] OR "fibrosis"[MeSH] OR "alanine transaminase"[MeSH] OR "aspartate aminotransferase"[MeSH] OR "alkaline phosphatase"[MeSH] OR "gamma glutamyltransferase"[MeSH] OR "triglycerides"[MeSH] OR "bilirubin"[MeSH] OR "prediabetes"[MeSH] OR "insulin resistance"[MeSH] OR "hyperinsulinism"[MeSH] OR "diabetes mellitus"[MeSH] OR "insulin"[MeSH] OR "metabolic syndrome"[MeSH] OR "glucose intolerance"[MeSH] OR "obesity"[MeSH] OR "carbohydrate metabolism"[MeSH] OR "fructosamine"[MeSH] OR "glucose tolerance test"[MeSH] OR "fatty liver"[Title/Abstract] OR "NAFLD"[Title/Abstract] OR "nonalcoholic steatohepa*"[Title/Abstract] OR "NASH"[Title/Abstract] OR "steatosis"[Title/Abstract] OR "liver enzymes"[Title/Abstract] OR "liver damage"[Title/Abstract] OR "liver injury"[Title/Abstract] OR "liver insufficiency"[Title/Abstract] OR "liver failure"[Title/Abstract] OR "liver fibrosis"[Title/Abstract] OR "MASLD"[Title/Abstract] OR "MASH"[Title/Abstract] OR "metabolic dysfunction-associated*"[Title/Abstract] OR "alanine amino*"[Title/Abstract] OR "ALP"[Title/Abstract] OR "ALT"[Title/Abstract] OR "gamma glutamyl trans*"[Title/Abstract] OR "GGT"[Title/Abstract] OR "alanine transaminase"[Title/Abstract] OR "aspartate aminotransferase"[Title/Abstract] OR "alkaline phosphatase"[Title/Abstract] OR "AST"[Title/Abstract] OR "triglyc*"[Title/Abstract] OR "cytokeratin 18"[Title/Abstract] OR "CK-18"[Title/Abstract] OR "CK18"[Title/Abstract] OR "total bili*"[Title/Abstract] OR "prediabetes"[Title/Abstract] OR "insulin"[Title/Abstract] OR "hyperinsulin*"[Title/Abstract] OR "diabetes mellitus"[Title/Abstract] OR "HbA1c"[Title/Abstract] OR "hemoglobin A1C"[Title/Abstract] OR "FA"[Title/Abstract] OR "fructosamine"[Title/Abstract] OR "GTT"[Title/Abstract] OR "glucose toler*"[Title/Abstract] )  AND  ( "high fructose corn syrup"[MeSH] OR "fructose"[MeSH] OR "high fructose corn syrup"[Title/Abstract] OR "HFCS*"[Title/Abstract] OR "fructose syrup"[Title/Abstract] OR "glucose-fructose"[Title/Abstract] OR "isoglucose"[Title/Abstract] OR "maize syrup"[Title/Abstract] OR "corn sweet*"[Title/Abstract] OR "crystalline fructose"[Title/Abstract] OR "fruit fructose"[Title/Abstract] OR "isolated fructose"[Title/Abstract] OR "corn syrup"[Title/Abstract] OR "fructose-glucose"[Title/Abstract]) |
| Web of Science | TS=(("fatty liver" OR "NAFLD" OR "nonalcoholic steatohepa*" OR "NASH" OR "steatosis" OR "liver enzymes" OR "liver damage" OR "liver injury" OR "liver insufficiency" OR "liver failure" OR "liver fibrosis" OR "alanine amino*" OR "ALP" OR "ALT" OR "gamma glutamyl trans*" OR "GGT" OR "alanine transaminase" OR "aspartate aminotransferase" OR "alkaline phosphatase" OR "triglyc*" OR "cytokeratin 18" OR "CK-18" OR "CK18" OR "MASLD" OR "MASH" OR "AST" OR "total bili*" OR "metabolic dysfunction-associated*" OR "prediabetes" OR "insulin" OR "hyperinsulin*" OR "diabetes mellitus" OR "HbA1c" OR "hemoglobin A1C" OR "FA" OR "fructosamine" OR "GTT" OR "glucose toler*")  AND  ( "high fructose corn syrup" OR "HFCS" OR "fructose syrup" OR "glucose-fructose" OR "isoglucose" OR "maize syrup" OR "corn sweet*" OR "crystalline fructose" OR "fruit fructose" OR "isolated fructose" OR "corn syrup" OR "fructose-glucose" ) ) |
| EMBASE | ( 'liver diseases'/exp OR 'fatty liver'/exp OR 'liver failure'/exp OR 'fibrosis'/exp OR 'alanine transaminase'/exp OR 'aspartate aminotransferase'/exp OR 'alkaline phosphatase'/exp OR 'gamma glutamyltransferase'/exp OR 'triglycerides'/exp OR 'bilirubin'/exp OR 'prediabetes'/exp OR 'insulin resistance'/exp OR 'hyperinsulinism'/exp OR 'diabetes mellitus'/exp OR 'insulin'/exp OR 'metabolic syndrome'/exp OR 'glucose intolerance'/exp OR 'obesity'/exp OR 'carbohydrate metabolism'/exp OR 'fructosamine'/exp OR 'glucose tolerance test'/exp OR 'fatty liver':ti,ab,kw OR 'NAFLD':ti,ab,kw OR 'nonalcoholic steatohepa*':ti,ab,kw OR 'NASH':ti,ab,kw OR 'steatosis':ti,ab,kw OR 'liver enzymes':ti,ab,kw OR 'liver damage':ti,ab,kw OR 'liver injury':ti,ab,kw OR 'liver insufficiency':ti,ab,kw OR 'liver failure':ti,ab,kw OR 'liver fibrosis':ti,ab,kw OR 'MASLD':ti,ab,kw OR 'MASH':ti,ab,kw OR 'metabolic dysfunction-associated*':ti,ab,kw OR 'alanine amino*':ti,ab,kw OR 'ALP':ti,ab,kw OR 'ALT':ti,ab,kw OR 'gamma glutamyl trans*':ti,ab,kw OR 'GGT':ti,ab,kw OR 'alanine transaminase':ti,ab,kw OR 'aspartate aminotransferase':ti,ab,kw OR 'alkaline phosphatase':ti,ab,kw OR 'AST':ti,ab,kw OR 'triglyc*':ti,ab,kw OR 'cytokeratin 18':ti,ab,kw OR 'CK-18':ti,ab,kw OR 'CK18':ti,ab,kw OR 'total bili*':ti,ab,kw OR 'prediabetes':ti,ab,kw OR 'insulin':ti,ab,kw OR 'hyperinsulin*':ti,ab,kw OR 'diabetes mellitus':ti,ab,kw OR 'HbA1c':ti,ab,kw OR 'hemoglobin A1C':ti,ab,kw OR 'FA':ti,ab,kw OR 'fructosamine':ti,ab,kw OR 'GTT':ti,ab,kw OR 'glucose toler*':ti,ab,kw )  AND  ( 'high fructose corn syrup'/exp OR 'fructose'/exp OR 'high fructose corn syrup':ti,ab,kw OR 'HFCS':ti,ab,kw OR 'fructose syrup':ti,ab,kw OR 'glucose-fructose':ti,ab,kw OR 'isoglucose':ti,ab,kw OR 'maize syrup':ti,ab,kw OR 'corn sweet*':ti,ab,kw OR 'crystalline fructose':ti,ab,kw OR 'fruit fructose':ti,ab,kw OR 'isolated fructose':ti,ab,kw OR 'corn syrup':ti,ab,kw OR 'fructose-glucose':ti,ab,kw) |
